# Study on the mesomechanical behavior and damage process of coal samples with hole defects under eccentric loading

**DOI:** 10.1371/journal.pone.0297994

**Published:** 2024-05-02

**Authors:** Lina Ge, Fu Yi, Tao Wang

**Affiliations:** 1 School of Vehicle and Transportation Engineering, Taiyuan University of Science and Technology, Taiyuan, China; 2 College of Architecture and Transportation, Liaoning Technical University, Fuxin, China; 3 School of Safety and Emergency Management Engineering, Taiyuan University of Technology, Taiyuan, China; University of Duhok, IRAQ

## Abstract

When using end shield shearer to recover end slope coal resources, the stability of the overlying rock slope of the end slope is controlled by leaving coal pillars. Due to the influence of the self weight of the overlying rock layer, the coal pillar will be subjected to eccentric loads, and the influence of eccentric loads needs to be considered in the design of the coal pillar size. With the help of PFC discrete element software, uniaxial compression tests were carried out on coal sample containing hole defects under different degrees of eccentric loads based on the calibration of micro mechanical parameters. The results show that the peak stress, cracking stress and dilatancy stress of coal sample decrease in a linear function law with the increase of load eccentricity coefficient. The evolution of the number of microscopic cracks during uniaxial compression under eccentric load can be divided into four stages: the calm stage before crack initiation I, the stable propagation stage II, the unstable propagation and penetration stage III, and the post failure stage IV. The distribution of macroscopic cracks is jointly influenced by the relative position of the loading area and the hole defect. When the hole defect is within the loading area, the hole plays a guiding role in the evolution of coal sample cracks, and the macroscopic crack runs through the edge of the loading area and the hole. When the hole defect is located outside the loading zone, the degree of eccentric load is large, weakening the guiding effect of the hole defect on the crack, and the macroscopic crack does not pass through the hole defect.

## 1 Introduction

Due to factors such as geological conditions and mining efficiency, the total amount of trapped coal under the slope of open-pit mines in China is over ten billion tons. The abandonment of this coal not only causes huge waste of resources, but also leaves significant safety hazards such as coal seam spontaneous combustion. The mining process of end shield shearers is an effective way to recover trapped coal resources in open-pit mines. Currently, it has been successfully applied in open-pit coal mines such as Trabula, Jinzhengtai, and Antaibao in China, and achieved significant economic benefits. When using the end shield shearer to mine the retained coal on the end wall, the stability of the overlying rock slope at the end is controlled by retaining supporting coal pillars between mining tunnels. Due to the presence of slopes, the coal pillar will be subjected to eccentric loads generated by the self weight of the overlying strata, which may exacerbate the deformation and failure of the coal body. The determination of the size of the coal pillar needs to consider the influence of eccentric loads. Therefore, conducting research on the damage and failure laws of coal pillars under eccentric loads has important theoretical guidance significance for the safe mining of end slope coal.

In mining and engineering construction, external disturbances and stress redistribution can cause defects such as pores and cracks in coal, rock, concrete, and other media. These defects, regardless of their size, can reduce the strength of the material, leading to material weakening [1]. Conducting research on the fracture process of surrounding rock around holes can help reveal the mechanism of rock damage and fracture, and provide a theoretical basis for evaluating the stability of excavation engineering surrounding rock [2, 3]. Wang et al. conducted uniaxial compression tests on granite specimens with central hole and edge defects, and found that the central hole and edge defects greatly weakened the strength and other mechanical properties of the specimens [4]. Wei et al. investigated the mechanical properties and cracking behavior of specimens with different pore shapes under uniaxial compressive load through laboratory experiments [5]. Xiao et al. conducted uniaxial acoustic emission experiments on rock samples with different positions and pore sizes, and analyzed the effects of different pore positions and diameters on the mechanical properties of rock samples [6]. Li et al. conducted research on the deformation and fracture mechanisms of coal and rock masses with different stress levels and crack angles, and analyzed the influencing factors of coal and rock deformation and failure and the mechanism of modification and strengthening [7]. Zhang et al. conducted uniaxial compression tests on porous fractured rocks and constructed a mechanical model to explain the formation mechanism of tensile cracks [8]. With the development of computer technology, numerical simulation methods are also an important means of studying the mechanical damage characteristics of materials at present [9]. Duan et al. used numerical simulation to study the failure process of brittle materials containing single and double pores under uniaxial compression conditions, and analyzed the influence of pore distribution on material strength and failure modes [10]. Zhao et al. conducted numerical and experimental research on the failure process of rock like materials with open hole defects, and proposed a failure strength model for brittle materials with open hole defects [11]. Tian et al. explored the mechanical properties and cracking behavior of perforated specimens under uniaxial compression load through indoor experiments, and conducted corresponding numerical simulations using PFC2D, capturing the force field distribution before and during cracking [12]. Ma et al. used Flac3D software and Digital Image Correlation (DIC) to simulate the internal stress distribution of intact red sandstone samples and various pore shaped samples, and studied the influence of cross structure on the mechanical properties and failure mechanism of rock materials [13]. Chen et al. conducted uniaxial compression tests on rock masses containing ten different types of pore defects using PFC2D software, and analyzed their failure behavior and mechanical properties [14]. Wang et al. used PFC2D software to establish coal rock samples with different dip angles of cracks and different positions of hole defects, and discussed the stress-strain characteristics and fracture evolution laws of coal rock with different crack holes [15].

In recent years, there have been some research results on the mechanical properties of rocks under eccentric loads. Zang and Yoon studied the effect of a form of eccentric load on the brittle failure characteristics of granite by conducting triaxial compression tests under eccentric load [16, 17]. Huang et al. conducted eccentric compression tests on concrete samples, and studied the effect of load eccentricity on concrete shrinkage and creep development, but did not study the instantaneous mechanical properties and damage characteristics of concrete samples under eccentric load [18]. Wang et al. studied the mechanical response characteristics of large-scale coal and rock masses under non-uniform load, Zhao et al. studied the internal microstructure and surface crack evolution of coal samples under asymmetric loading, but the research results did not involve the coupling effect of the rich primary pore and crack structure of raw coal and non-uniform load on the damage law of coal and rock [19, 20].

As can be seen from the above research results that scholars have achieved rich research results on the mechanical properties and damage mechanisms of coal and rock containing defects under uniformly distributed loads, but there are few reports on the damage and failure process of coal and rock with hole defects under eccentric loads. This article uses PFC numerical simulation to study the micro fracture process of coal sample with pore defects under eccentric load uniaxial compression, and compares and verifies the simulation results with indoor test results. The research results have important theoretical guidance significance for the safe mining of end face coal.

## 2 Establishment of PFC calculation model and calibration of mesoscopic parameters

### 2.1 Numerical model

The existing researches show that in the PFC discrete element method, the two-dimensional numerical simulation results can well reflect the mechanical properties and damage and failure laws of coal and rock samples. Therefore, the paper adopts a two-dimensional plane stress model to study the mechanical characteristics and damage evolution law of coal samples with hole defects under eccentric load.

The constitutive characteristics of materials in PFC are simulated through contact constitutive models, including contact stiffness models, sliding models, and bonding models, and there are two types of bonding models: contact bonding model and parallel bonding model. The parallel bonding model is a parallel bonding on a finite size (rectangular or circular cross-section), which can obtain both a force and a moment, and the parallel bonding theory has been explained in detail in the research results of Potyondy and Cundall [21, 22].

**Fig 1:**
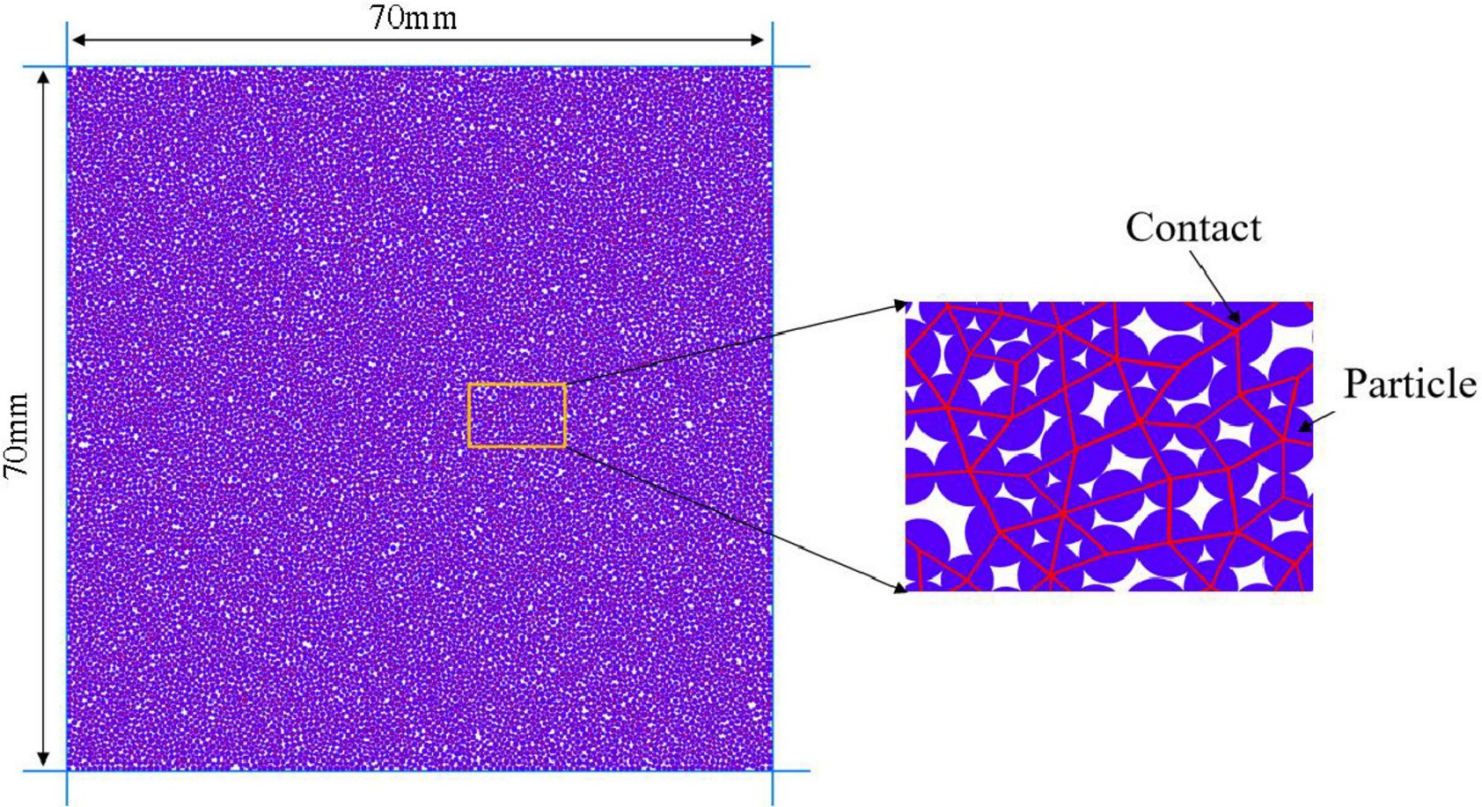
Model of numerical calculation.

This article simulates the mechanical behavior of rocks by setting up a parallel bonding model, as shown in Fig 1. Before establishing the PFC model, a calculation area needs to be defined, set to 6 times the model width and 1.6 times the model height to ensure sufficient space to move the wall for eccentric loading. The size of the established model is consistent with the coal sample size in the indoor experiment, which is 70 mm × 70 mm, the PFC program creates a sample by filling particles into the wall body During PFC calculation process, and the total number of particles required for model establishment is 12033, the minimum particle diameter is set to 0.2 mm and the particle size ratio is set to 1.66. After filling, the sample needs to be pre compressed by moving the upper and lower walls, as well as the left and right walls. After the preloading is completed, remove the left and right walls, and finally load the sample by moving the upper and lower walls [23–26].

### 2.2 Calibration of mesoscopic parameters

The values of mesoscopic parameters in numerical calculations determine the deformation and failure characteristics of the model. Due to the dispersion of particles, the true mechanical parameters of the samples obtained from indoor experiments cannot be directly assigned to the particles and contact elements. At present, the “trial and error method” is often used to determine the values of mesoscopic parameters, which continuously adjusting the values of mesoscopic parameters to conduct simulation experiments and comparing the mechanical parameters and macroscopic failure modes of the simulated samples with the indoor test results, until a set of mesoscopic parameters is found that make the mechanical parameter values of the two not significantly different and the failure modes consistent, it is considered that the mechanical characteristics and damage failure laws of coal samples can be simulated using this set of mesoscopic mechanical parameters [27, 28].

The calibration of mesoscopic parameters was carried out using the results of uniaxial compression tests on intact raw coal samples with a side length of 70mm. The loading equipment is an electro-hydraulic servo universal testing machine with a range of 300 KN, using the displacement loading mode and setting the loading speed to 0.1 mm/min. In the numerical simulation, the model is established with the same size as the indoor test, and the eccentric loading of the model is achieved through the movement of the upper and lower walls. The displacement loading mode is also used in numerical simulation. In order to save the running time of numerical simulation, while ensuring quasi-static loading, the moving speed of the loaded wall is set to 0.05 m/s.

**Fig 2:**
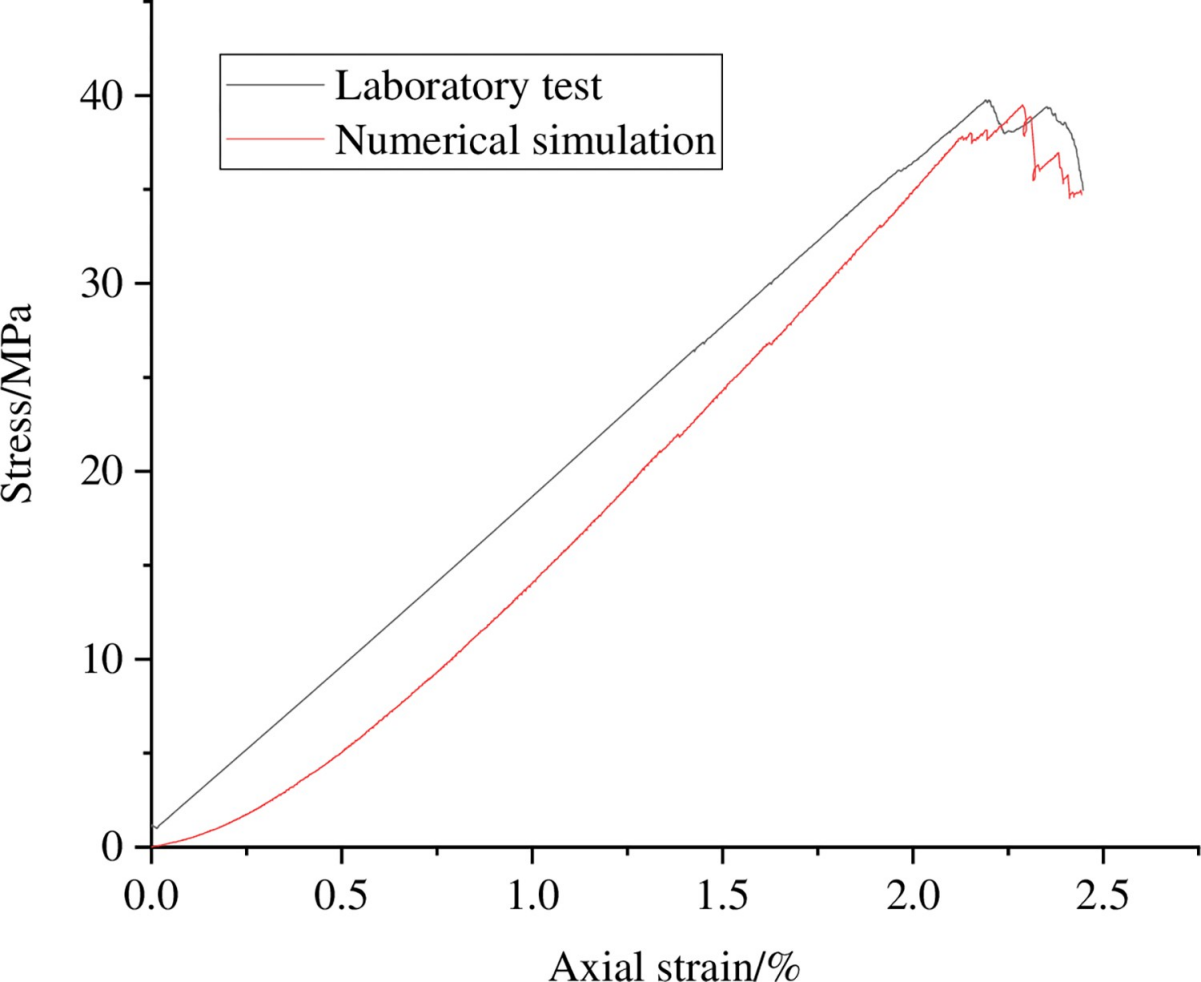
Laboratory test and numerical simulation stress-strain curves of coal sample.

The stress-strain curves obtained from indoor experiments and numerical simulations are shown in Fig 2. Table 1 summarizes the mechanical parameter values of coal sample obtained from indoor experiments and numerical simulations, among which Poisson’s ratio is obtained from the triaxial compression test of coal samples in the indoor experiment and by measuring the axial and transverse strains of the elastic stage specimen through circular monitoring in numerical simulation. From Fig 2 and Table 1, it can be seen that the peak stress, elastic modulus, and Poisson’s ratio of coal sample obtained from numerical simulation are not significantly different from the indoor test results.

**Table 1:** Mechanical parameters of coal sample obtained from laboratory test and numerical simulation.

Item	Peak stress(MPa)	Elastic modulus(GPa)	Poisson’s ratio
Test result	39.50	1.884	0.23
Simulation results	38.77	1.813	0.25
Deviation	1.85%	3.77%	8.6%

**Fig 3:**
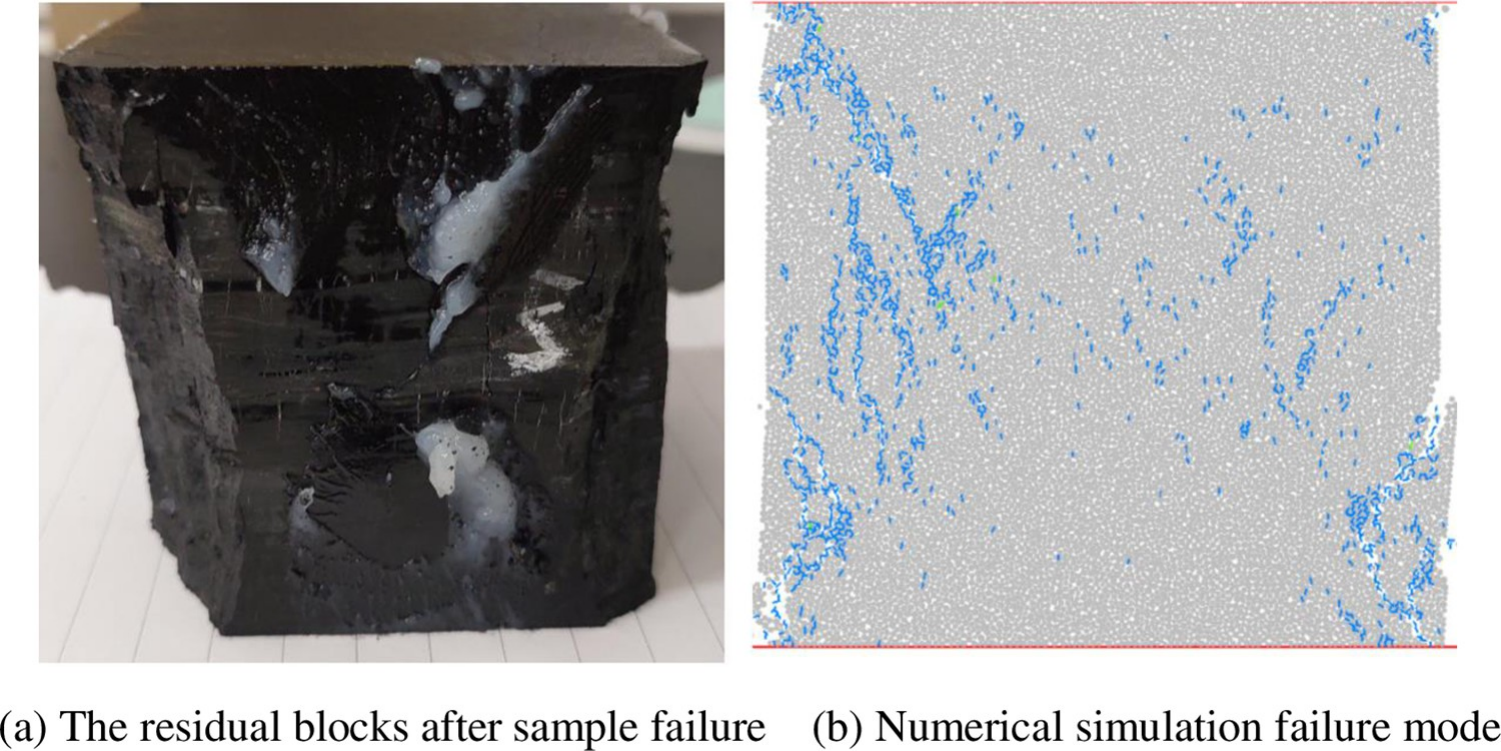
Failure modes of coal sample in laboratory test and numerical simulation. (a) The residual blocks after sample failure (b) Numerical simulation failure mode.

Fig 3 shows the failure modes of coal sample obtained from indoor experiments and numerical simulations. Due to the instantaneous disintegration of the raw coal sample in the indoor experiment, we use the residual blocks to determine the failure mode of the coal sample. Based on the shape of the residual blocks after sample failure in Fig 3(A), it is determined that the failure mode of the raw coal is X-type shear failure. From Fig 3(B), we can see that the failure mode of the sample obtained from numerical simulation is basically consistent with the indoor test results. Therefore, this set of mesoscopic parameter values can be used to simulate the damage and failure process of coal sample under different loading conditions in indoor experiments. The mesoscopic parameter values for successful calibration are shown in Table 2.

**Table 2:** Micromechanical parameters used in PFC2D simulation.

Mesoscopic parameters	Value
The minimum particle radius *R*_min_/mm	0.25
Ratio of maximum to minimum particle radius	1.66
Particle density *ρ*/(kg/m^3^)	2500
Friction coefficient between particles *μ*	0.3
Elastic modulus between particles *E*_*c*_/GPa	1.5
Contact bonding stiffness ratio k_n_/k_s_	2.82
Parallel bonding radius coefficient *λ*	1
Elastic modulus of parallel bonding ${\bar{E}}_{c}$/GPa	1.5
Parallel bonding stiffness ratio ${\bar{k}}_{n}/{\bar{k}}_{s}$	2.82
porosity	0.14
Parallel bonding tensile strength ${\bar{\sigma }}_{c}$/MPa	27
Parallel bonding cohesion $\bar{c}$/MPa	33
Parallel bonding internal friction angle $\bar{\varphi }$/(°)	30

## 3 Simulation of uniaxial compression of coal sample with hole defects under eccentric load

### 3.1 Definition of load eccentricity coefficient

To facilitate the representation of the degree of load eccentricity, the load eccentricity coefficient is defined as the ratio of the non loading area to the loading area.


$$I_{c}=1‐\frac{S_{f}}{S}$$ (1)

In the formula, *I*_c_ is the eccentricity coefficient, S_f_ is the area directly affected by the load, and S is the surface area of the sample.

According to Eq (1), the corresponding load eccentricity coefficients for loading areas of 1S/4, 2S/4, 3S/4, and S are 0.75, 0.5, 0.25, and 0, respectively.

### 3.2 Simulation scheme for uniaxial compression under eccentric load

**Fig 4:**
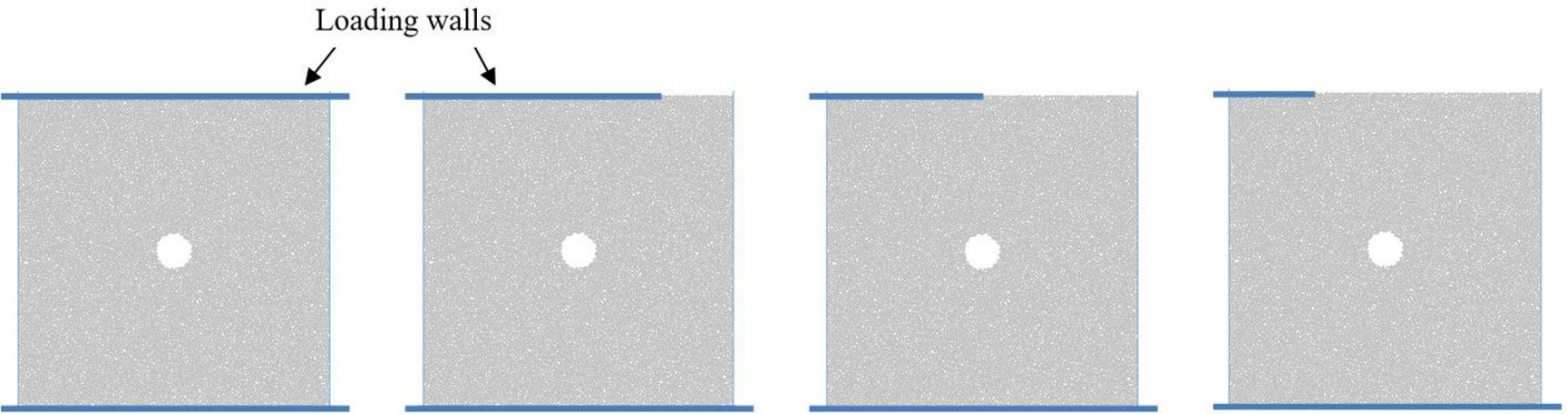
Uniaxial compression simulation scheme of coal sample with hole defect under eccentric loading.

The numerical simulation of eccentric loading method is shown in Fig 4. The loading of the sample is achieved through the movement of the upper and lower walls, and the movement speed value of the wall is consistent with the calibrated parameter in simulation. The measurement of axial and transverse strains is achieved by laying measurement circles.

### 3.3 Influence of eccentric load on mechanical parameters of coal sample

**Fig 5:**
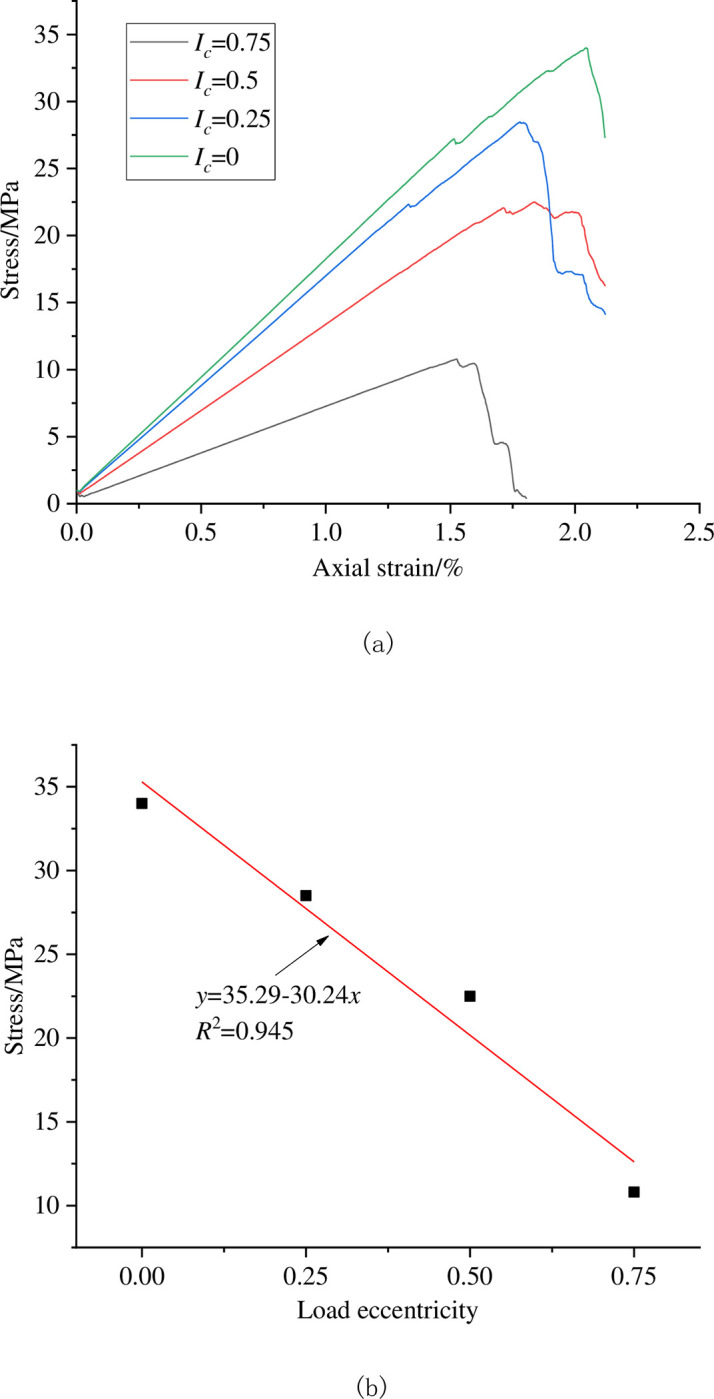
Stress and strain characteristics of coal sample under eccentric load. (a) The stress-strain curve (b) Relationship between peak stress and eccentricity coefficient.

In order to analyze the influence of eccentric load on the mechanical parameters of coal sample, The stress-strain values of coal sample under different degrees of eccentricity loading measured by the measurement circle monitoring method are shown in Fig 5.

As shown in Fig 5, under different load eccentricity coefficients, the stress-strain curves of coal sample exhibit the same characteristics. With the increase of axial strain, the stress of coal sample gradually increases, and with the increase of eccentricity coefficient, the peak stress of coal sample gradually decreases. The load eccentricity coefficient and peak stress approximately follow the linear function law, that is, the greater the load eccentricity, the more likely the coal samples are to be damaged.

The initiation stress is an important characteristic value of rock strength and an important indicator of the initiation and propagation of microcracks within the rock [29]. Potyondy proposed a method for determining the cracking stress of rocks based on the number of microscopic cracks through research, which first determines the number of cracks *m*_c_ in the sample at the peak stress, and then finds the stress corresponding to 1% of cracks *m*_c_ as the simulated cracking stress value *σ*_ci_ [30]. In addition, the appearance of the reverse bending point of the volume strain curve indicates the expansion of the rock volume, which is the result of the intersection and connection of cracks. The stress corresponding to the reverse bending point of the volume strain curve is the dilatancy stress *σ*_cd_.

**Fig 6:**
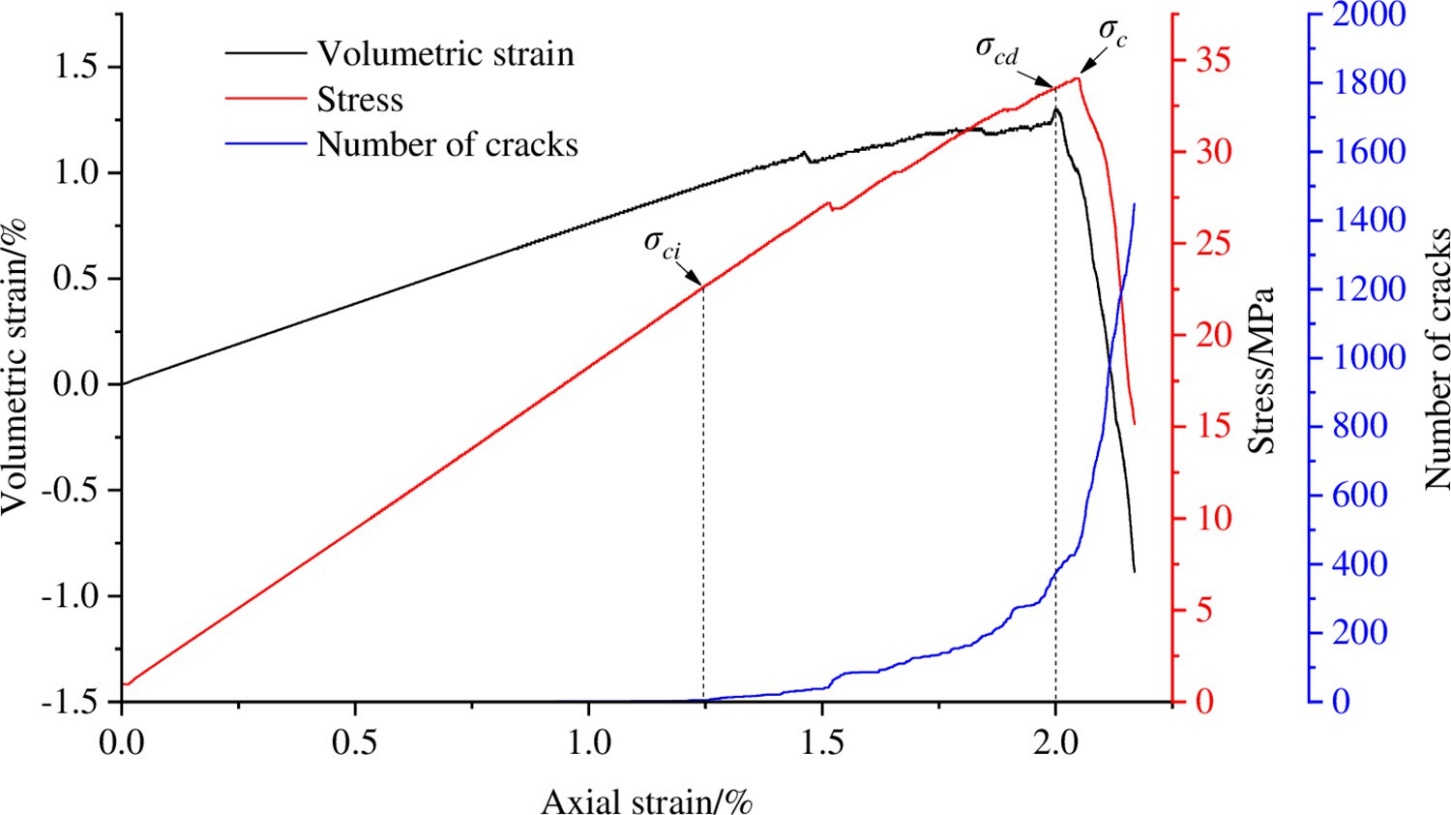
Calibration of cracking stress and dilatancy stress of coal sample when *I*_c_ = 0.

**Fig 7:**
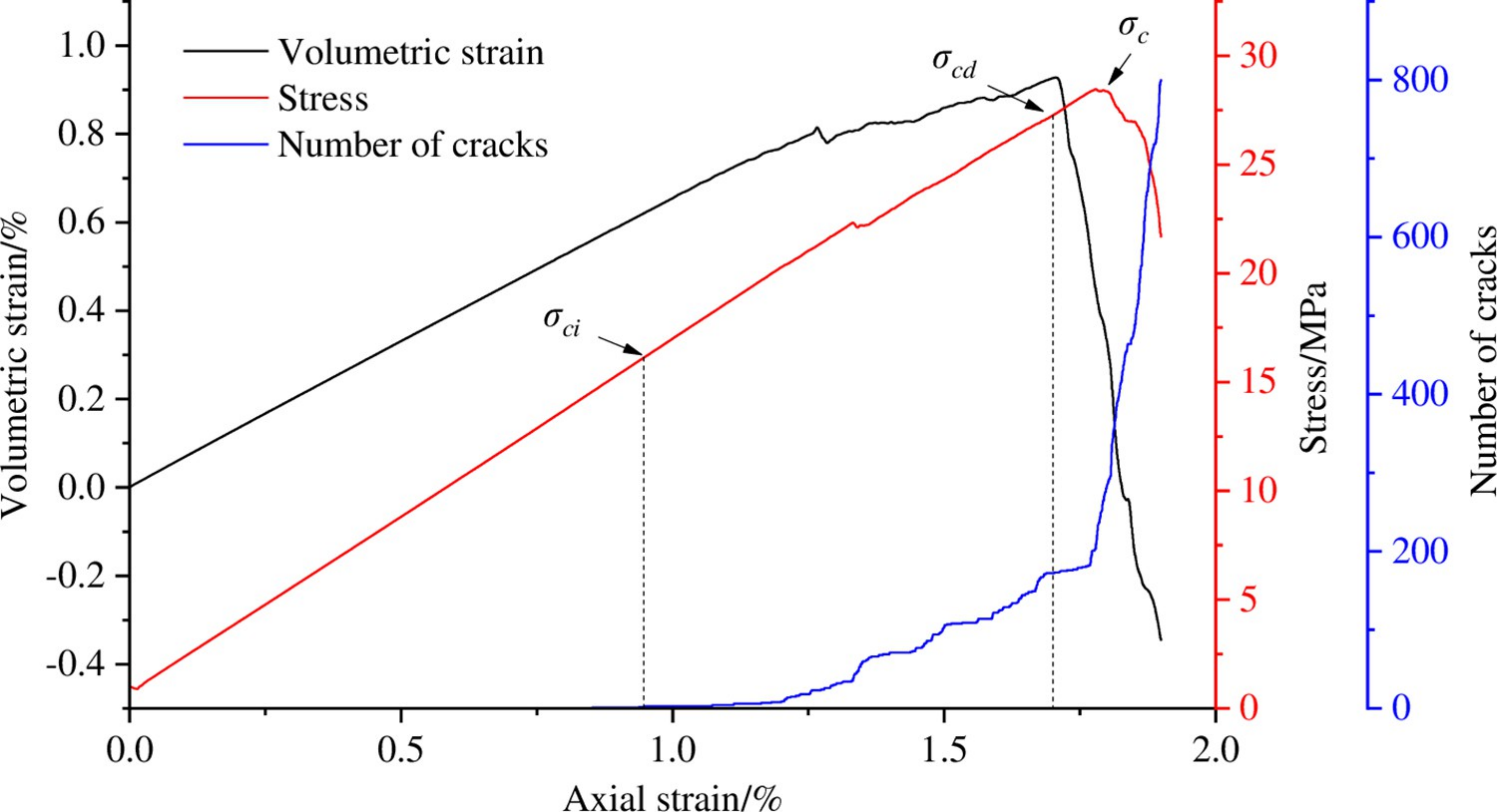
Calibration of cracking stress and dilatancy stress of coal sample when *I*_c_ = 0.25.

**Fig 8:**
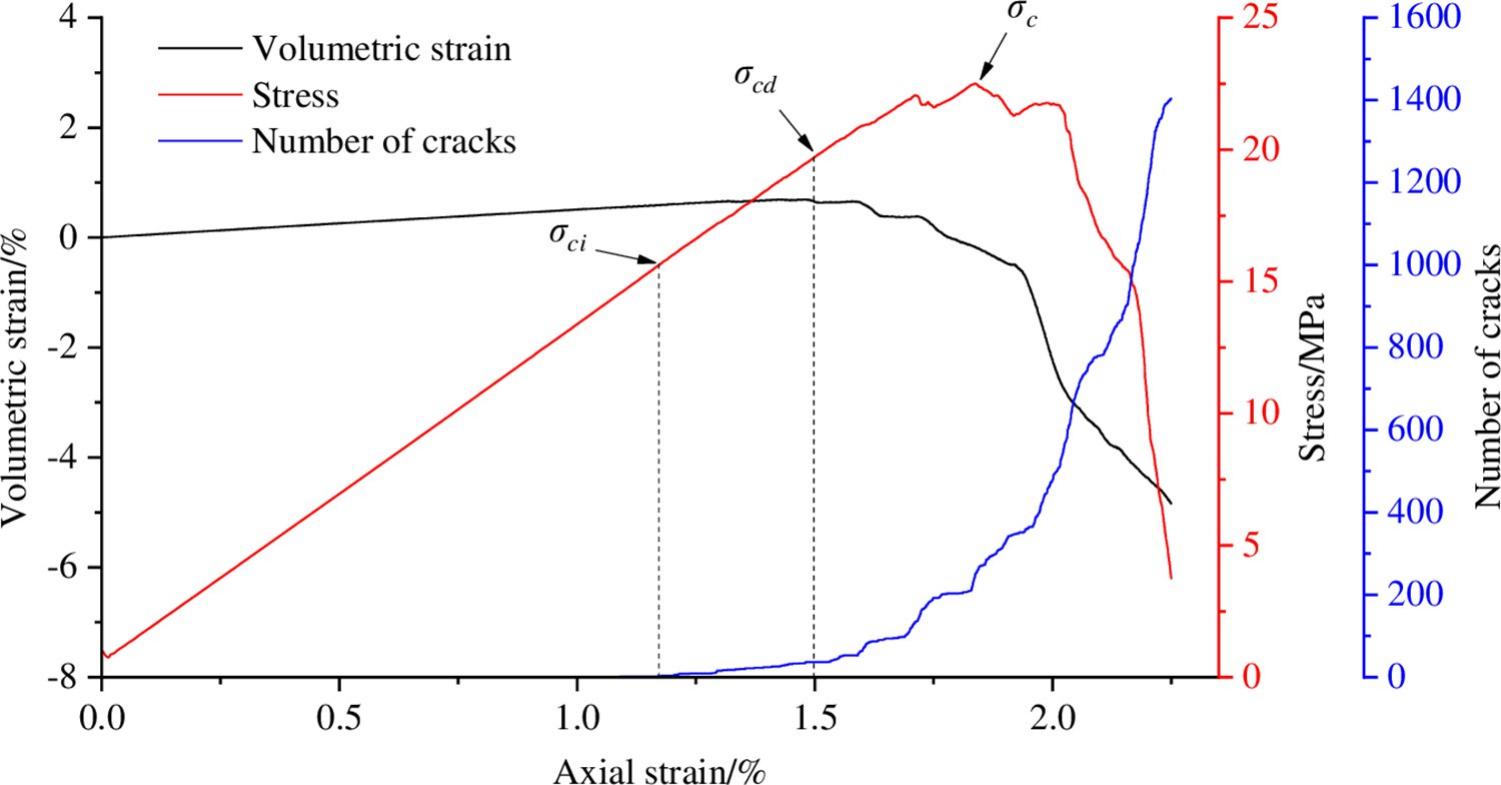
Calibration of cracking stress and dilatancy stress of coal sample when *I*_c_ = 0.5.

**Fig 9:**
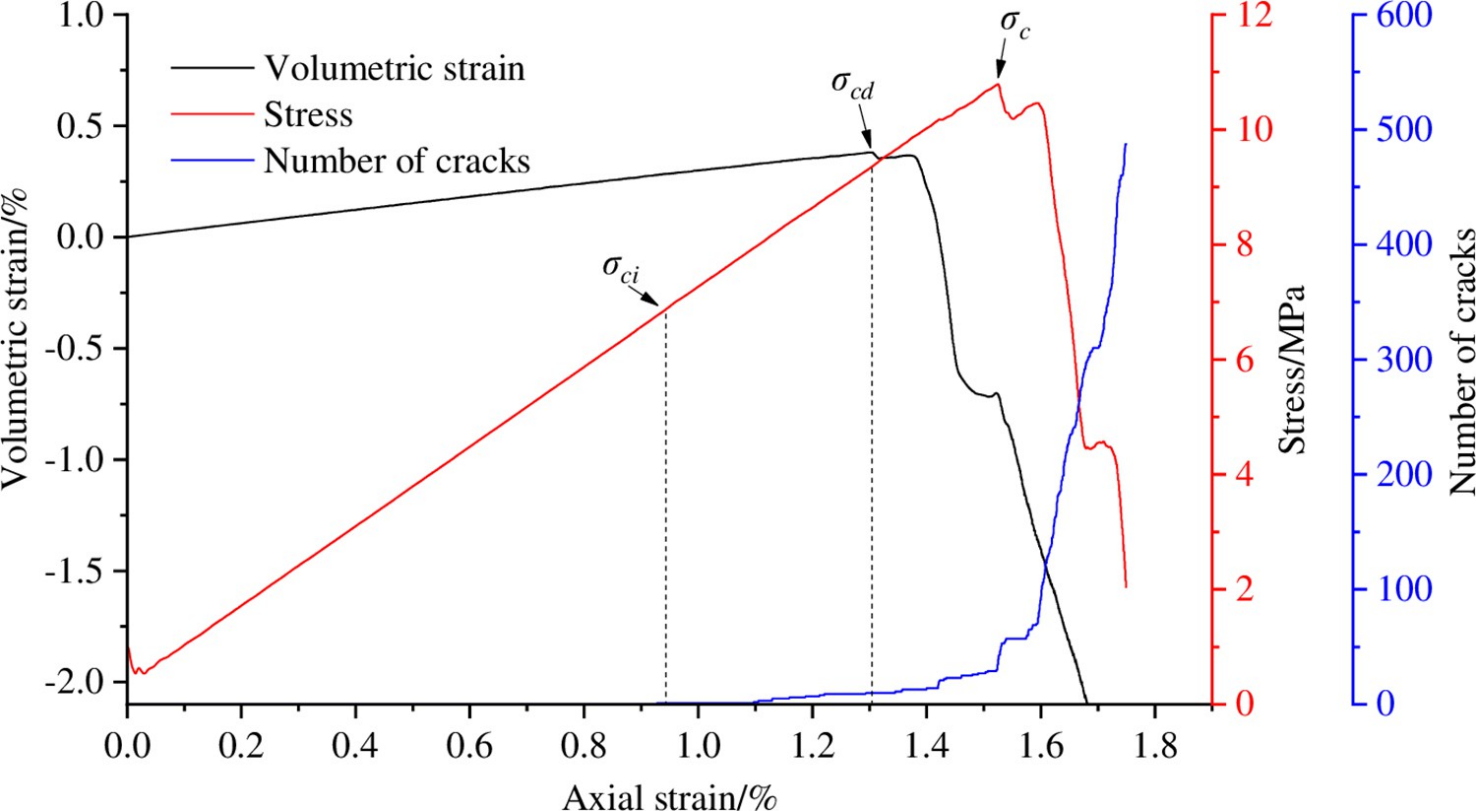
Calibration of cracking stress and dilatancy stress of coal sample when *I*_c_ = 0.75.

With the help of the above method, the cracking stress and dilatancy stress of coal sample are calibrated in the process of eccentric loads of different degrees, as shown in Figs 6–9. Table 3 shows the cracking stress and dilatancy stress values of coal sample under different degrees of eccentric loads as well as the strain values corresponding to the cracking stress and dilatancy stress.

**Table 3:** Cracking stress value and dilatancy stress value of coal sample.

*I*_c_	Cracking stress /*σ*_ci_ (MPa)	Cracking strain /*ε*_c_	Dilatancy stress /*σ*_cd_ (MPa)	Dilatancy strain*/ε*_cd_	Peak stress/*σ*_c_ (MPa)
0	22.6	0.0124	33.4	0.0200	34.00
0.25	16.12	0.0095	27.25	0.0170	28.40
0.5	15.65	0.0117	19.69	0.0150	22.49
0.75	6.86	0.0094	9.37	0.0131	10.78

**Fig 10:**
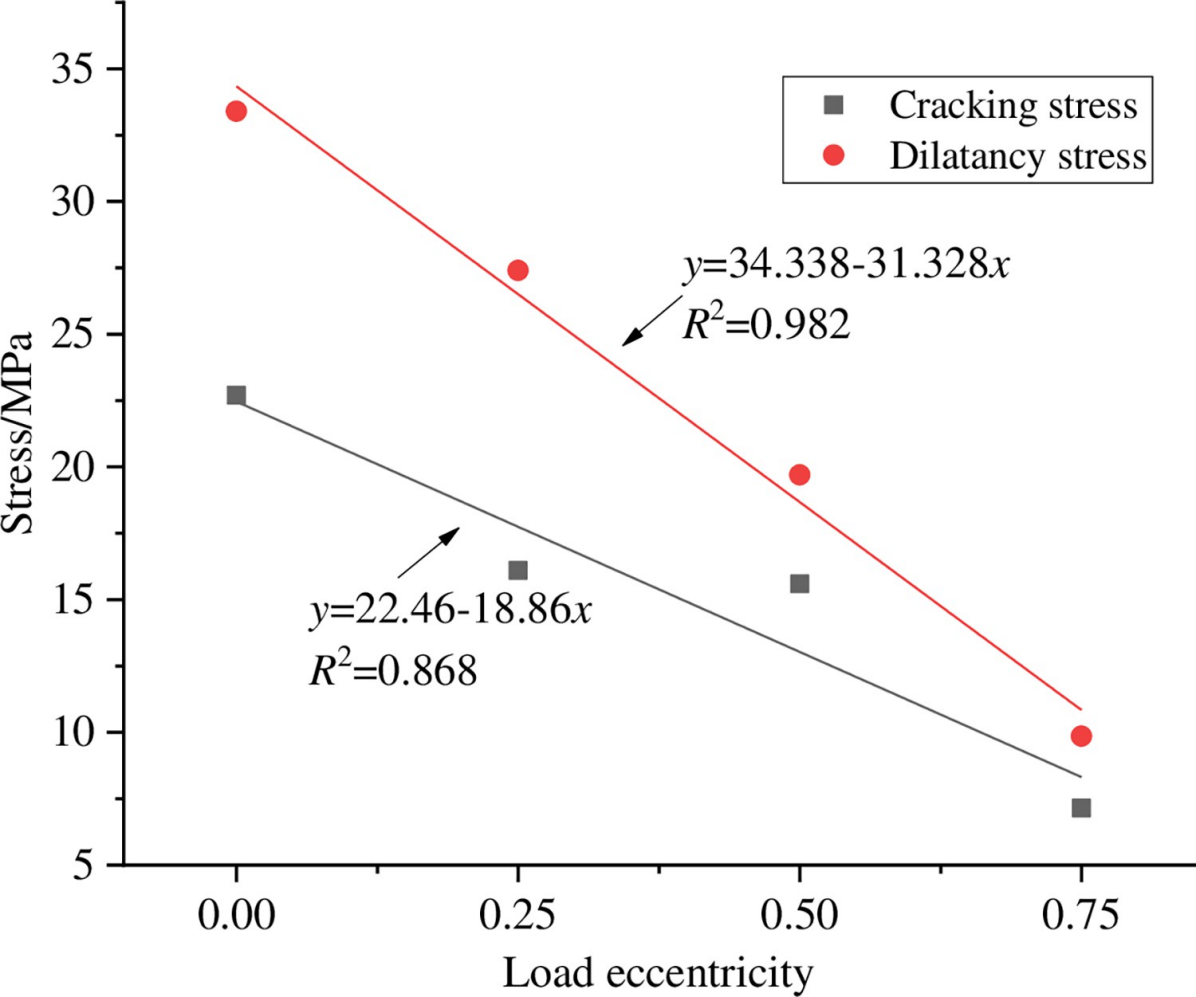
Cracking stresses and dilatancy stresses of coal sample under different load eccentricity.

In order to study the effect of eccentricity coefficient on the cracking stress and dilatancy stress of coal sample, the data in Table 3 are fitted, and the results are shown in Fig 10. It can be seen from Fig 10 that with the increase of eccentricity coefficient, the cracking stress and dilatancy stress of coal sample gradually decrease, and both are in linear function relationship with the load eccentricity coefficient, which indicates that with the increase of load eccentricity, micro cracks in coal sample are more prone to initiation and failure.

### 3.4 The influence of eccentric load on the macroscopic failure mode of coal sample

**Fig 11:**
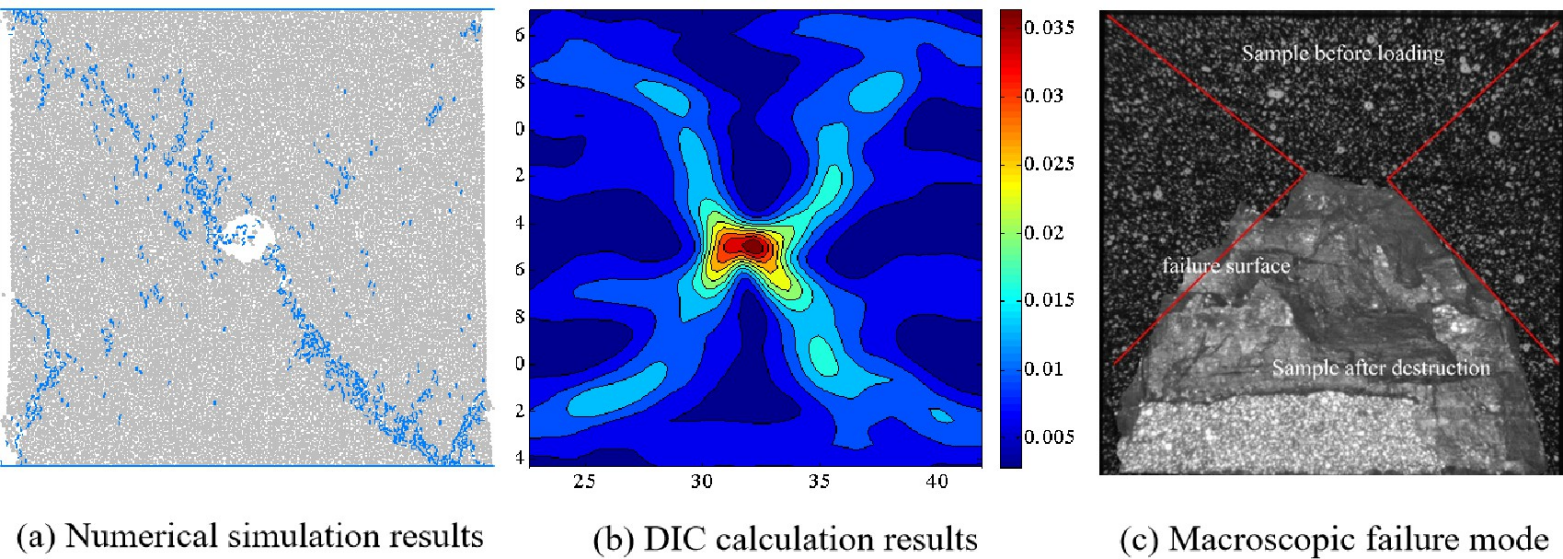
Comparison of numerical simulation failure mode and indoor test results when *I*_c_ = 0.

**Fig 12:**
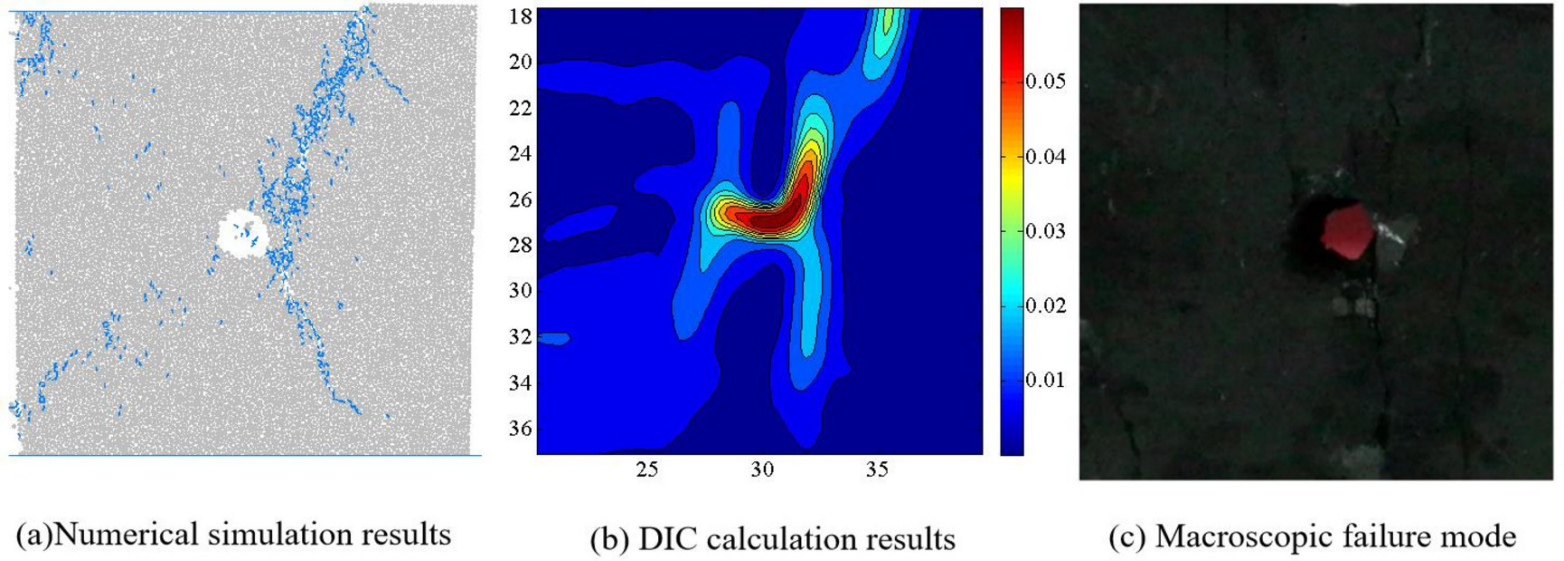
Comparison of numerical simulation failure mode and indoor test results when *I*_c_ = 0.25.

**Fig 13:**
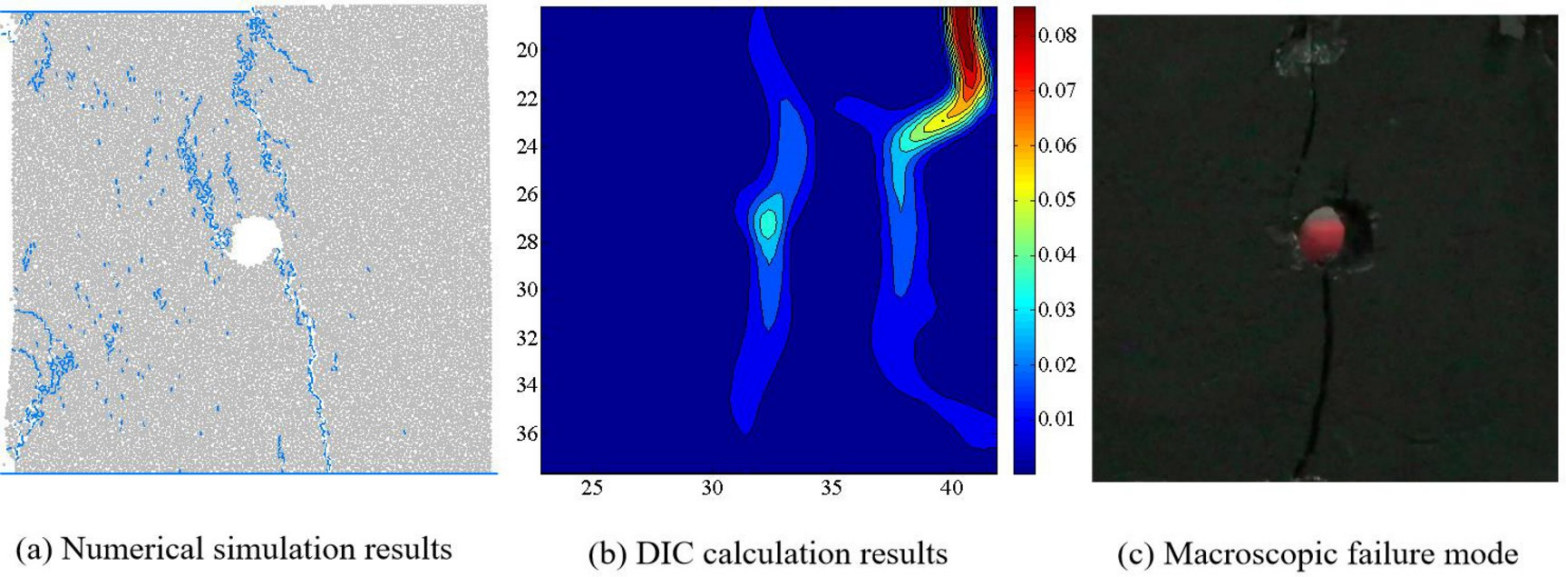
Comparison of numerical simulation failure mode and indoor test results when *I*_c_ = 0.5.

**Fig 14:**
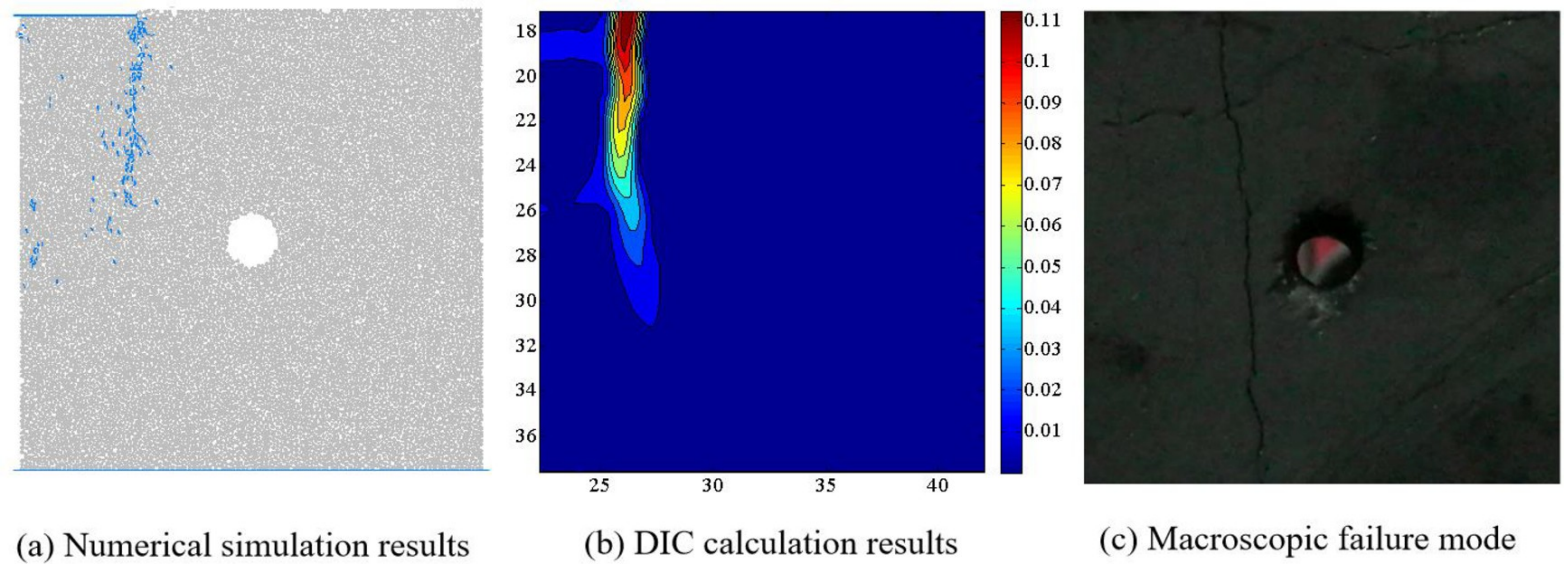
Comparison of numerical simulation failure mode and indoor test results when *I*_c_ = 0.75.

Figs 11–14 show the comparison results of macroscopic failure modes of coal sample obtained from numerical simulation and indoor experiments under different degrees of eccentric load. From Figs 11–14, it can be seen that the macroscopic failure modes of coal sample obtained from numerical simulation are roughly consistent with the indoor test results. Under uniform load, coal sample mainly undergo X-type shear failure, while under eccentric load, coal sample undergo macroscopic shear failure.

Zhao et al. have conducted a systematic study on the crack evolution laws of intact coal samples under four eccentric loading conditions. Based on the research results of Zhao et al. on the internal microstructure and surface crack evolution of intact coal samples under asymmetric loading, we conclude that: the distribution of macroscopic cracks obtained from numerical simulation are influenced by the relative position of the loading area and the hole defect [19]. When the hole defect is within the loading area, i.e. *I*_c_ = 0, 0.25 and 0.5, the hole plays a guiding role in the evolution of coal rock cracks, and the macroscopic cracks run through the edge of the loading area and the hole. When the hole defect is located outside the loading zone, i.e. *I*_c_ = 0.75, due to the large degree of eccentric load, the guiding effect of the hole defect on the crack is weakened, and the macroscopic crack does not pass through the hole defect.

### 3.5 Effect of eccentric load on the number of microscopic cracks in coal sample

**Fig 15:**
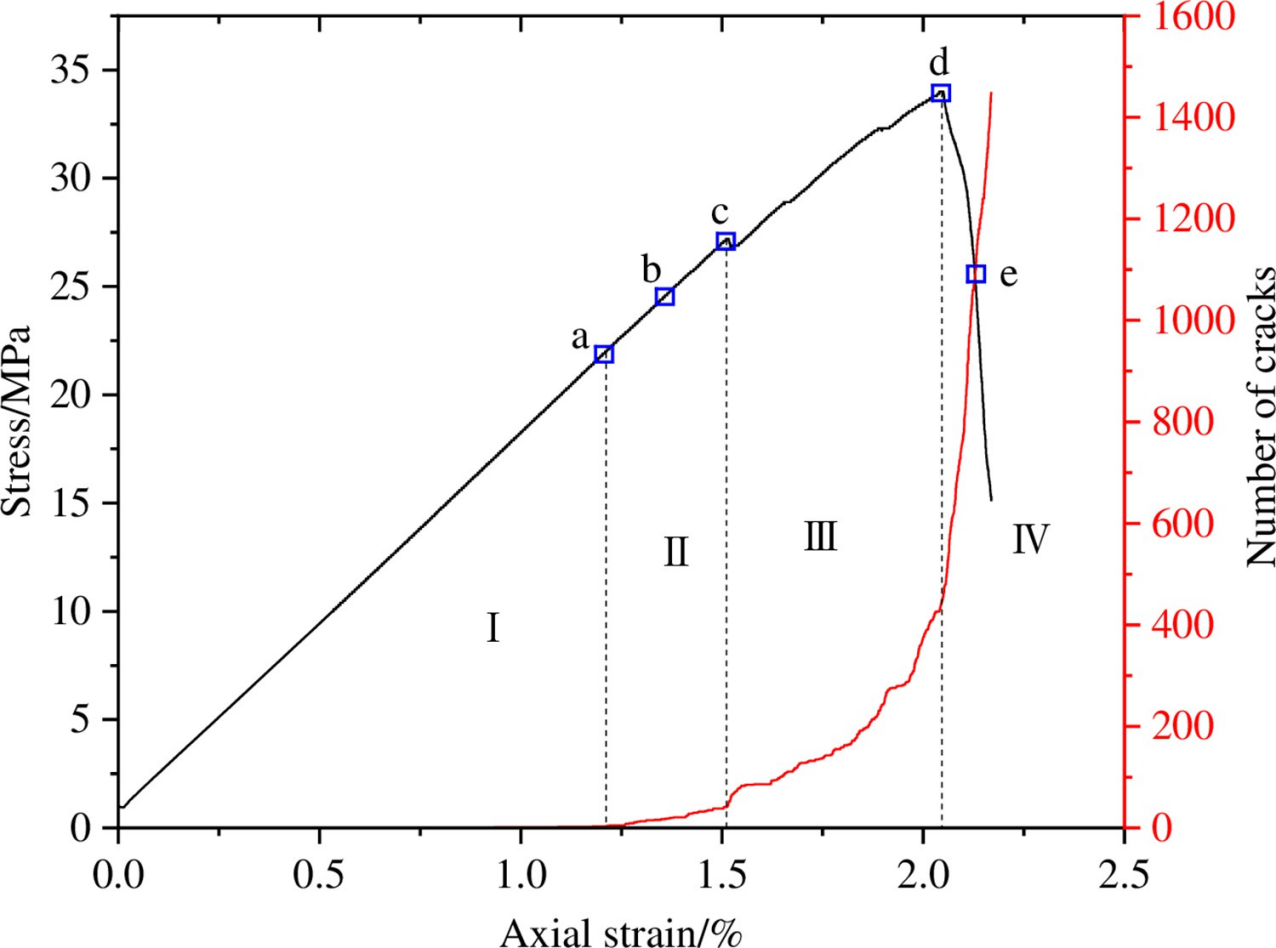
Crack number evolution curve when *I*_c_ = 0.

**Fig 16:**
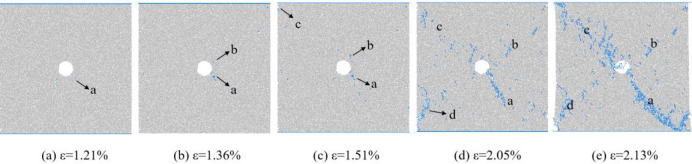
Meso crack evolution law when *I*_c_ = 0.

The changes in the number of micro cracks are recorded during the numerical calculation process, and Fig 15 shows the evolution relationship of the number of cracks with increasing axial strain under uniform load conditions. As shown in Fig 15, the number of cracks in coal sample gradually increases with the increase of axial strain, which can be divided into four stages: the calm stage I before crack initiation, the stable propagation stage II, the unstable propagation and penetration stage III, and the post failure stage IV. Select typical moments at different stages of crack propagation and analyze the propagation and evolution process of microscopic cracks, as shown in Fig 16. From Fig 16, it can be seen that during the stable crack propagation stage II, the micro cracks are scattered and concentrated near the hole defects. As the axial strain increases, the sample enters the stage of unstable crack propagation and penetration III, during which the number of cracks rapidly increases and the distribution tends to be centralized, mainly distributed in the connection area between the sample end angle and the hole defect. At the peak moment, the microscopic cracks intersect with each other, forming macroscopic cracks. Based on the distribution pattern of macroscopic cracks, it can be inferred that the sample undergoes macroscopic shear failure.

**Fig 17:**
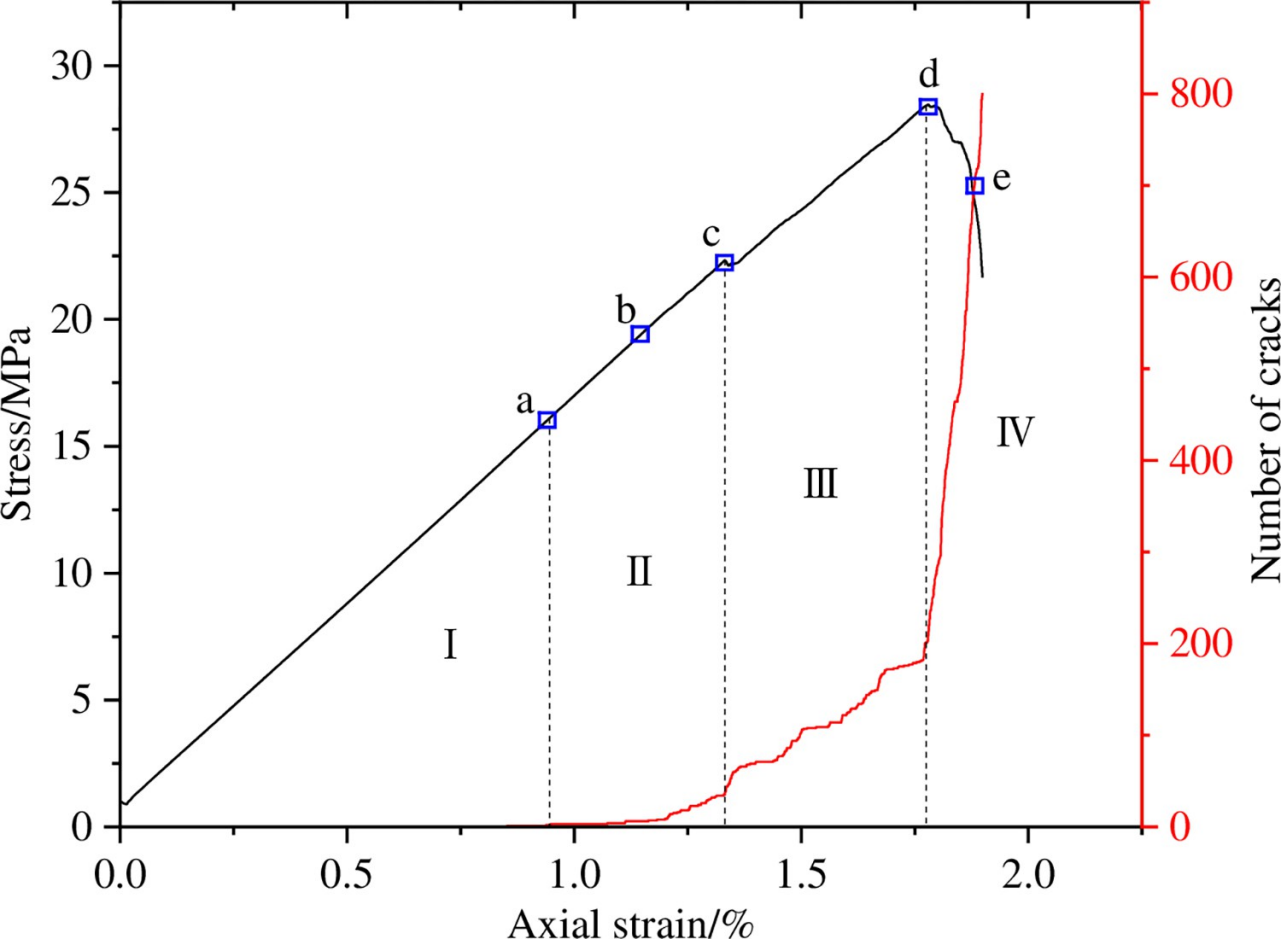
Crack number evolution curve when Ic = 0.25.

Figs 17, 19 and 21 show the evolution curves of the number of coal rock cracks under varying degrees of eccentric loading. From Figs 17, 19 and 21, it can be seen that the evolution law of the cracks number of coal sample under different degrees of eccentric load are consistent with that of uniformly distributed load. The evolution process of crack number can also be divided into four stages, namely the calm stage I before crack initiation, the stable crack propagation stage II, the unstable crack propagation and penetration stage III, and the post failure stage IV.

**Fig 18:**
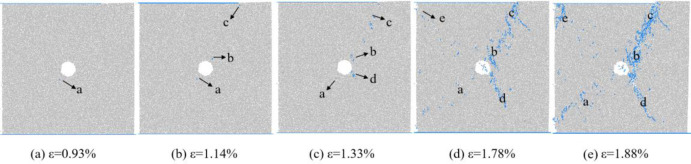
Meso crack evolution law when Ic = 0.25.

Figs 17 and 18 show the number and distribution evolution of micro cracks at *I*_c_ = 0.25. From Figs 17 and 18, it can be seen that the stress concentration at the hole causes the crack to first crack in this area a. In stage II of stable crack propagation, the number of micro cracks around the pores slowly increases, Cracks c are generated at the end of the loading plate due to stress concentration and gradually propagate along the shear action zone between the load action zone and the non action zone. The crack enters the stage of unstable propagation and penetration III, and the number of cracks increases rapidly. There are two concentrated zones in the distribution of cracks. On the one hand, cracks are concentrated in the shear zone between the load action area and the non action area, and shift towards the direction of the hole defect. On the other hand, similar to uniformly distributed loads, there are also a large number of cracks distributed in the connecting area between the end of the sample and the hole defect. As the axial strain increases, the cracks in the above two areas gradually evolve and connect, leading to the failure of the sample. The above analysis indicates that when *I*_c_ = 0.25, the degree of load eccentricity is not strong, and the sample failure can be divided into two parts: uniaxial compression part and eccentric load part. The distribution of cracks generated in the uniaxial compression area is the same as that of uniformly distributed loads, while cracks generated in the eccentric load area are distributed between the load action area and the non action area, and deflect towards the hole defect area. The combined effect of eccentric load and pore defects leads to the failure of the specimen.

**Fig 19:**
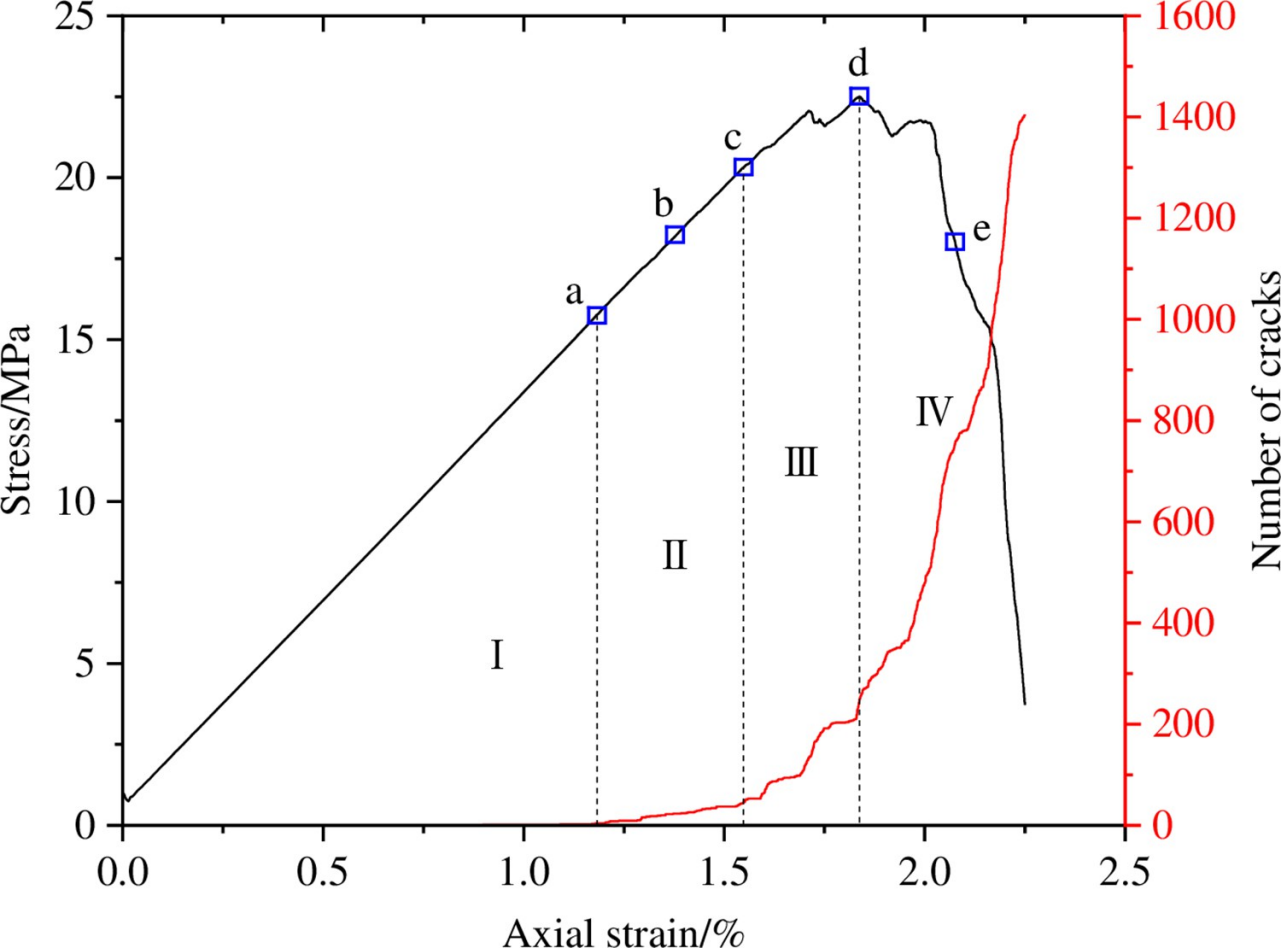
Crack number evolution curve when Ic = 0.5.

**Fig 20:**
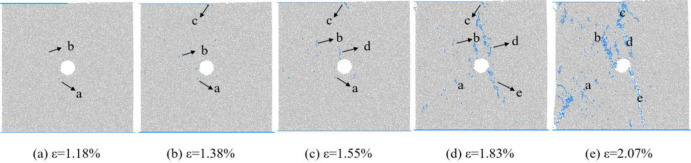
Meso crack evolution law when Ic = 0.5.

Figs 19 and 20 show the number and distribution evolution of micro cracks at *I*_c_ = 0.5. From Figs 19 and 20, it can be seen that after the calm period I, the crack still first cracks from the stress concentration position a at the hole defect, and then cracks c also appear at the end of the loading plate. As the axial strain increases, the crack b propagates towards both the upper end of the sample and the hole defect. In the stage of unstable crack propagation, crack d and crack c propagate in the opposite direction, forming macroscopic cracks distributed in the shear zone, crack e propagates from the hole defect to the bottom of the specimen. Due to the fact that the hole defect is located between the load action zone and the non action zone, the dominant crack formed during specimen failure is also located in this region, and is approximately parallel to the loading direction.

**Fig 21:**
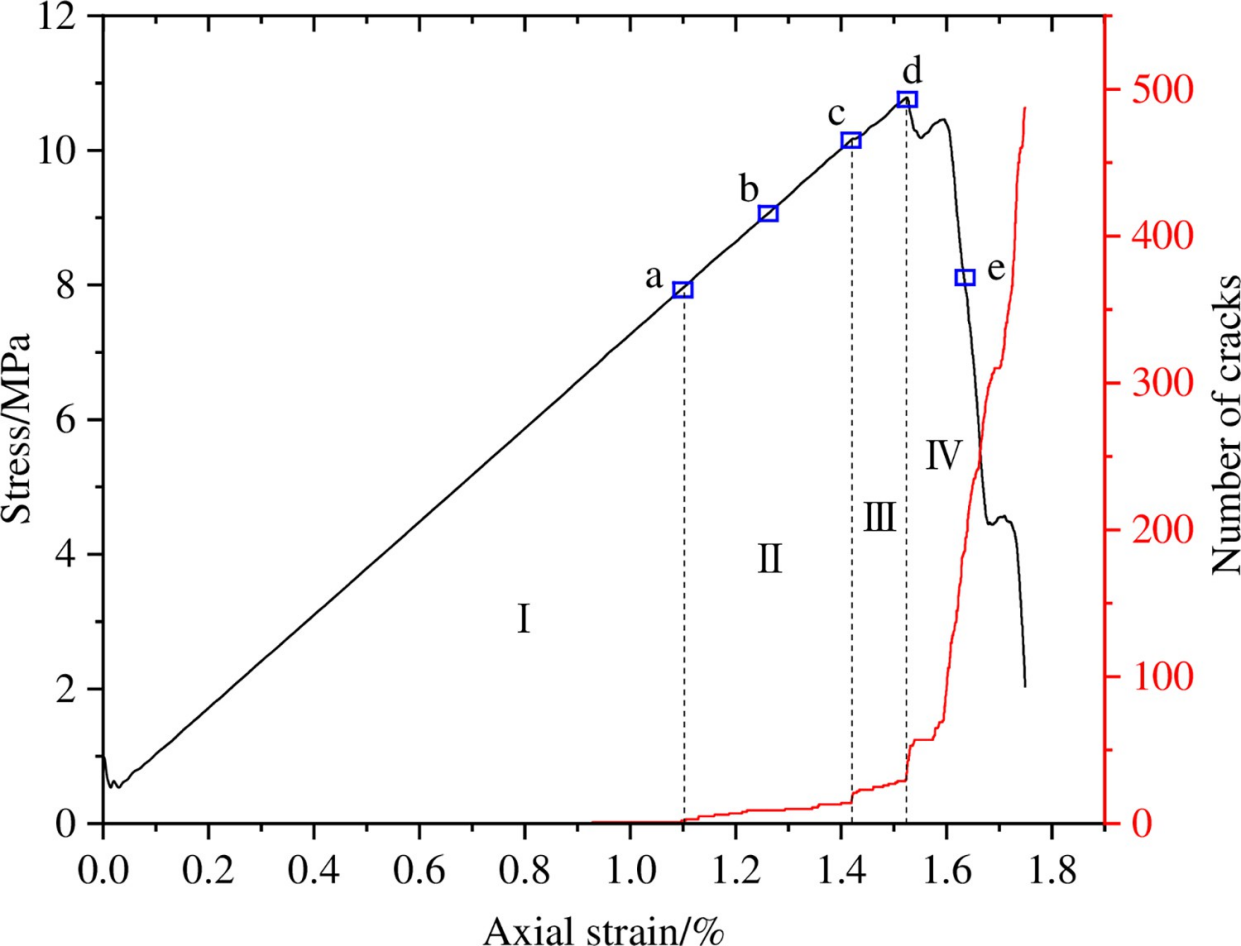
Crack number evolution curve when Ic = 0.75.

**Fig 22:**
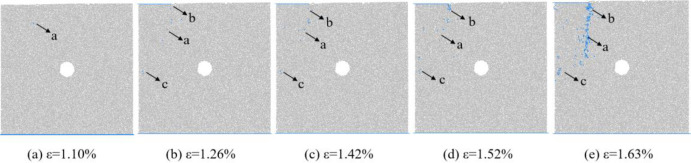
Meso crack evolution curve when Ic = 0.75.

Figs 21 and 22 show the number and distribution evolution of micro cracks at *I*_c_ = 0.75. From Figs 21 and 22, it can be seen that after the calm period, cracks a are generated between the load action zone and the non action zone, and cracks b are generated at the end of the loading plate. As the axial strain increases, cracks c are generated at the middle edge of the sample, and the number of cracks continues to increase in the connection areas of a, b, and c. The final penetration forms macroscopic cracks, and the sample undergoes failure. The main control crack formed is located in the shear zone between the load action zone and the non action zone, deflecting towards the load action zone. At this point, the hole defect is located outside the load action zone, with a large degree of load eccentricity. The guiding effect of the hole defect on crack propagation is not reflected, and the direction of crack evolution is not deviated towards the direction of the hole defect.

Combine Zhao et al.’s research on the mechanical properties of intact coal and rock masses under asymmetric loads with the above analysis, it can be seen that the initiation and propagation direction of micro cracks in coal sample under eccentric load are jointly affected by the degree of load eccentricity and pore defects [19]. When the hole defect is located within the range of the load action zone, the hole defect guides the propagation of the crack, and the macroscopic crack formed after the sample failure is penetrated by the hole defect. When the hole defect is located outside the load action zone, the degree of load eccentricity is large, which limits the guiding effect of the hole defect on crack propagation. The crack propagates under eccentric load and is not affected by the hole defect.

## 4 Conclusions

1. The peak stress, crack initiation stress and dilatancy stress of coal sample are gradually reduced with the increase of load eccentricity coefficient, and they are in linear function with the load eccentricity coefficient.
2. Under uniform load, the coal sample mainly undergoes X-type shear failure, while under eccentric load, the coal sample undergoes macroscopic shear failure.
3. The evolution of the number of micro cracks during uniaxial compression under eccentric load can be divided into four stages: the calm stage I before crack initiation, the stable propagation stage II, the unstable propagation and penetration stage III, and the post failure stage IV.
4. The distribution of macroscopic cracks obtained from numerical simulation is controlled by the influence of the relative position between the loading area and the hole defect. When the hole defect is within the loading zone, the hole plays a guiding role in the evolution of coal rock cracks, and the macroscopic crack runs through the edge of the loading zone and the hole. When the hole defect is located outside the loading zone, the degree of eccentric load is large, weakening the guiding effect of the hole defect on the crack, and the macroscopic crack does not pass through the hole defect.

## Supporting information

S1 Data (ZIP)
